# Portuguese Advance Directives-a twist against futility? A cross sectional study

**DOI:** 10.1590/1516-3180.2022.0537.R2.201023

**Published:** 2024-03-25

**Authors:** Catarina Sampaio Martins, Rui Nunes

**Affiliations:** I MD, MSc. Physician, Serviço de Medicina Paliativa do Centro Hospitalar de Trás-os-Montes e Alto Douro, Vila Real, Portugal; PhD student, Faculdade de Medicina, Universidade do Porto, Porto, Portugal.; II PhD. Cathedratic Professor, Departamento de Medicina da Comunidade, Informação e Decisão em Saúde Faculdade de Medicina, Universidade do Porto, Porto, Portugal.

**Keywords:** Advance Directives, Medical Futility, Palliative Care, Legislation as topic, Health Literacy, End-of-life, Last Will, Public Law, Portugal Legislation, Decision-making

## Abstract

**BACKGROUND:**

Advance Directive documents allow citizens to choose the treatments they want for end-of-life care without considering therapeutic futility.

**OBJECTIVES:**

To analyze patients’ and caregivers’ answers to Advance Directives and understand their expectations regarding their decisions.

**DESIGN AND SETTING:**

This study analyzed participants’ answers to a previously published trial, conceived to test the document’s efficacy as a communication tool.

**METHODS:**

Sixty palliative patients and 60 caregivers (n = 120) registered their preferences in the Advance Directive document and expressed their expectations regarding whether to receive the chosen treatments.

**RESULTS:**

In the patient and caregiver groups, 30% and 23.3% wanted to receive cardiorespiratory resuscitation; 23.3% and 25% wanted to receive artificial organ support; and 40% and 35% chose to receive artificial feeding and hydration, respectively. The participants ignored the concept of therapeutic futility and expected to receive invasive treatments. The concept of therapeutic futility should be addressed and discussed with both the patients and caregivers. Legal Advanced Directive documents should be made clear to reduce misinterpretations and potential legal conflicts.

**CONCLUSION:**

The authors suggest that all citizens should be clarified regarding the futility concept before filling out the Advance Directives and propose a grammatical change in the document, replacing the phrase “Health Care to Receive / Not to Receive” with the sentence “Health Care to Accept / Refuse” so that patients cannot demand treatments, but instead accept or refuse the proposed therapeutic plans.

**TRIAL REGISTRATION:**

ClinicalTrials.gov ID NCT05090072

**URL:**

https://clinicaltrials.gov/ct2/show/NCT05090072.

## INTRODUCTION

Given the increasing growth in scientific knowledge and the advancements in medical treatments, patients’ autonomy regarding end-of-life care must be promoted.^1^

Advance Directives have emerged as self-determination documents enabling patients to participate in end-of-life decision-making when they can no longer manifest their wishes.^2^

In Portugal, Advance Directives were legalized in 2012 (Law 25/2012 of 16th July).^3^ This legislative norm enables citizens to nominate a health surrogate and register their preferences on a Living Will^4^ document with a five-year expiry date. Every adult citizen capable of providing free and informed consent can register their Living Will with the proper legal authority of the Ministry of Health.^3^

The Portuguese Advance Directives document^4^ comprises a two-step formulation of a tick-box type. In the first section (clinical situations to apply Advance Directives), citizens choose clinical scenarios where they wish to apply their end-of-life preferences. In the second section (Healthcare to receive or not), they register their Advance Directives by ticking the boxes according to their preferences (**Table 1**).^4^

**Table 1.** Demographic characteristics of the population

	Patients	Caregivers
**Age (years)**, mean ± SD	70.6 ± 13.2	58.6 ± 13.5
**Gender**, n (%)		
Male	32 (53.3%)	16 (26.7%)
Female	28 (46.7%)	44 (73.3%)
**Education Level**, n (%)		
Illiterate	7 (11.7%)	1 (1.7%)
Knows how to write and read	9 (15.0%)	4 (6.7%)
Primary School	30 (50.0%)	15 (25.0%)
Middle School	11 (18.3%)	17 (28.3%)
High School	3 (5.0%)	12 (20.0%)
University	0 (0.0%)	11 (18.3%)
**Religion**, n (%)		
Catholic	59 (98.3%)	55 (91.7%)
Jehovah’s Witness	1 (1.7%)	2 (3.3%)
Agnostic	0 (0.0%)	1 (1.7%)
Other	0 (0.0%)	2 (3.3%)

SD = standard deviation.

Through decades, the Advance Directives were implemented and promoted as a legal document that allowed patients to refuse the treatments they considered unacceptable for themselves, protecting them from therapeutic futility and medical obstination.^5^ The Portuguese law reinforces that idea by stating that the citizens can choose not to receive cardiorespiratory resuscitation or invasive organ support, among others.^3^ However, the Advance Directives’ documents^4^ are silent regarding the concept of medical futility and do not raise patients’ and citizens’ awareness on this issue, although they should, to prevent inadequate expectations and future disagreements or litigations between patients, caregivers, and the healthcare personnel. Therefore, when patients do not tick the boxes of refusal of blood product administration, cardiopulmonary resuscitation, or invasive organ support, they probably assume they will receive those treatments, ignoring that in some clinical circumstances, those procedures might be considered futile and contrary to “legis artis” and, as such, not be accomplished.

## OBJECTIVES

This study aimed to analyze the answers to the Advance Directive formulary^4^ of a population of patients receiving palliative care and their caregivers and to understand their decisions when filling the document.

## METHODS

### Design and setting

This study analyzed the answers of a group of participants enrolled in the DAVPAL (Advance Directives in Palliative Care) trial (available on ClinicalTrials.gov, ID NCT05090072), which was conceived to test the use of the Advance Directive document as an instrument to promote better concordance between patients and caregivers regarding end-of-life care.

All patients were referred to the Palliative Medicine Service between September 2018 and September 2019, and their caregivers were invited to participate. Participants were enrolled in the study if the following inclusion criteria were fulfilled: adult patients who agreed to participate in the DAVPAL Trial, had no cognitive impairment, and could understand and speak Portuguese.

This study adhered to the ethical procedures outlined in the Declaration of Helsinki. The study was approved by the Ethics Committee of the Centro Hospitalar de Trás-os-Montes e Alto Douro on June 18, 2018 (Doc no. 245/2018), and all participants provided written informed consent to participate in the trial.

We asked 60 patients receiving palliative care and 60 caregivers (n = 120) to fill in the Portuguese Advance Directive formulary and express their preferences and expectations regarding the treatments for their end-of-life care.

The Portuguese model of Advance Directives^4^ was used to register the participants’ preferences. All participants filled in the documents individually and expressed their expectations when deciding whether to tick each sentence.

## RESULTS

The demographic characteristics of the study population are shown in **Table 1**. We observed that the patients were older than the rest of the participants and the caregiver group included a higher proportion of females. The patient group had low literacy levels and most participants were Catholic, as expected in Portugal.

**Table 2** shows the Portuguese Advance Directives’ formulary.^4^ To be officially valid, these documents must be registered on the platform—National Registration of the Living Will^6^ (RENTV) by a governmental health employee.

**Table 2.** Portuguese Advance Directives (Living Will)^4^

CLINICAL SITUATIONS TO APPLY THE ADVANCE DIRECTIVES When I am incapable of expressing my will, because of my mental or physical health situation, and one or more of the following hypotheses occur: ◻ Diagnosis of incurable and terminal disease ◻ No expectable recovery, according to state of art ◻ Unconsciousness with irreversible neurologic or psychiatric disease complicated by respiratory, renal, or cardiac disfunction ◻ Other _________________________________________________
HEALTH CARE TO RECEIVE / NOT TO RECEIVE Therefore, I manifest my clear and unequivocal will of: ◻ Not receive cardiorespiratory resuscitation ◻ Not be submitted to invasive and artificial organ support ◻ Not be submitted to artificial feeding and hydration for delaying the occurrence of natural death ◻ Participate in experimental studies or investigation trials ◻ Not be submitted to experimental treatments ◻ Not be submitted to experimental studies or investigation trials ◻ Interrupt previously consented experimental treatments or investigation trials participation ◻ Not authorize blood and derivates transfusions ◻ To receive palliative care and minimal oral or subcutaneous hydration ◻ To be administered effective and necessary pain killers and other symptom control drugs ◻ To receive spiritual assistance when invasive life support is about to end ◻ Be accompanied by the following person __________ when invasive life support is about to end

All participants chose the three clinical scenarios in the first section of the document (clinical situations where the Advance Directives apply), and their answers differed only in the second section of the document (Healthcare to Receive / Not to Receive); therefore, only the answers in this section will be presented and analyzed.

We enhanced the answers to the first three sentences of the second part of the document.^4^ They concern treatments that might be considered futile in the end-of-life period of most patients facing the scenarios of the first section, and particularly when they have terminal diseases for which they are receiving palliative care. The participants’ decisions are presented in **Table 3**.

**Table 3.** Participants’ answers to the Advance Directives^4^ (healthcare to receive or not)

Based on the scenarios previously described,	PALLIATIVE PATIENTS (n = 60)		CAREGIVERS (n = 60)	

I manifest my clear and unequivocal will of:	Selected	Did Not Select	Selected	Did Not Select
	n (%)	n (%)	n (%)	n (%)
Not to receive cardiorespiratory resuscitation	42 (70.0%)	**18 (30.0%)**	46 (76.7%)	**14 (23.3%)**
Not be submitted to invasive and artificial organ support	46 (76.7%)	**14 (23.3%)**	45 (75.0%)	**15 (25.0%)**
Not be submitted to artificial feeding and hydration for delaying the occurrence of natural death	36 (60.0%)	**24 (40.0%)**	39 (65.0%)	**21 (35.0%)**
Participate in experimental studies or investigation trials	38 (63.3%)	22 (36.7%)	37 (61.7%)	23 (38.3%)
Not be submitted to experimental treatments	22 (36.7%)	38 (63.7%)	21 (35.0%)	39 (65.0%)
Not be submitted to experimental studies or investigation trials	21 (35.0%)	39 (65.0%)	21 (35.0%)	39 (65.0%)
Interrupt previously consented experimental treatments or investigation trials participation	19 (31.7%)	41 (68.3%)	16 (26.7%)	44 (73.3%)
Not authorize blood and derivates transfusions	19 (31.7%)	41 (68.3%)	23 (38.3%)	37 (63.3%)
To receive palliative care and minimal oral or subcutaneous hydration	59 (98.3%)	1 (1.7%)	60 (100.0%)	0 (0.0%)
To be administered effective and necessary pain killers and other symptom control drugs	59 (98.3%)	1 (1.7%)	60 (100.0%)	0 (0.0%)
To receive spiritual assistance when invasive life support is about to be ended	51 (85.0%)	9 (15.0%)	48 (80.0%)	12 (20.0%)
Be accompanied by the following person when invasive life support has ended _______________________	54 (90.0%)	6 (10.0%)	45 (75.0%)	15 825.0%)

As expected, in the circumstances of terminal disease such as no expected recovery, or irreversible neurological or psychiatric disease with vital organ dysfunction, most participants refused invasive treatments and CPR. In the patients’ and caregivers’ groups, respectively, 70.0% and 76.7% chose “Not to receive cardiorespiratory resuscitation,” 76.7% and 75% chose “Not to receive artificial organ support,” and 60.0% and 65% chose “Not to receive artificial feeding and hydration only to delay the natural death occurrence”.

However, a considerable number of patients and caregivers did not refuse to undergo invasive procedures. When asked about their decisions, all the participants expressed that they expected to receive these treatments. In the patient group, despite facing incurable, progressive, or fatal diseases for which they were receiving palliative treatment, 30% wanted to receive cardiorespiratory resuscitation, 23.3% wanted to receive invasive and artificial organ support, and 40% chose to receive artificial feeding and hydration to delay the occurrence of natural death.

A significant number of participants in the caregiver group also chose not to refuse these invasive treatments when faced with previously described clinical scenarios. In this group, 23,3% chose cardiorespiratory resuscitation, 25% chose artificial organ support, and 35% chose artificial feeding and hydration to delay natural death. Similarly, as in the patient group, all these caregivers mentioned that they wanted and expected to receive these treatments if their heart or any vital organ stopped, despite being in a clinical situation of incurable and fatal diseases.

The main reasons for choosing artificial life support were religious and the concept that life must be preserved at any cost, as “miracles happen.” Spontaneous commentaries such as “my faith helps me not to give up,” “while there is life, there is hope,” and “only God knows when it is time to die” were used to justify the decisions made. One participant from the patients’ group even claimed that he had previously been on artificial life support and “woke up” to explain his choice of receiving invasive measures to postpone death. However, in both groups, most patients and caregivers chose to receive palliative care and symptom control drugs.

All participants ignored that cardiorespiratory resuscitation, invasive and artificial organ support, and artificial measures to delay natural death, might not be considered good practice in the previously chosen clinical scenarios and for most patients in palliative care. The participants were unaware of the concept of medical utility. They believed that the Advance Directives gave them a choice of treatment, regardless of whether they were indicated in their clinical situation or considered futile. They all stated that they expected to receive these invasive treatments, as they knew that the healthcare staff had to comply with the Advance Directives’ content.

## DISCUSSION

In palliative care patients, all invasive treatments must be weighted and pursued only when physicians have strong evidence that they will benefit the patients more than harm them. However, when patients are unfamiliar with the futility concept and choose to receive invasive treatments in Advance Directive documents, they may create unrealistic expectations of receiving them, even when they are considered futile.

Although the Portuguese population’s health literacy has improved,^7^ some patients and citizens may be unaware of what is considered “good medical practice,” and legal considerations may emerge if they understand that their autonomy and self-determination are not being accomplished. Health professionals are crucial in raising patient awareness regarding these issues, although the concept of futility is challenging for patients, physicians, and families to define and perceive differently.

The initial concept of futility as a non-beneficial, ineffective, and inappropriate treatment (Ethics Committee of the Society of Critical Care Medicine, 1997)^8^ evolved to other definitions such as “an intervention that is unlikely to restore, maintain, or enhance a life that the patient can be aware of”^9,10^ or “interventions with a meagre chance of benefitting the patient (quantitative futility), and interventions that will produce benefits with shallow quality (qualitative futility).”^11^

Morata^12^ proposed a consensus definition for futility as “interventions or procedures which do not achieve meaningful recovery of the primary ailment based on the patient’s and multidisciplinary teams’ healthcare goals, yet a latent sense of hope often underlies the situation and patient condition.”^12^

Unfortunately, futile treatments are performed worldwide and are well-documented in the literature. In a systematic review of non-beneficial treatments in hospitals at the end-of-life,^13^ that included 1,213,171 participants across 10 different countries, the most frequently reported situations were non-beneficial ICU admissions (10% prevalence), newly initiated or ongoing chemotherapy (33% prevalence), cardiorespiratory resuscitation for terminal patients (28,1% prevalence), death in the ICU and on a hospital ward, or after initiating aggressive treatment (58% prevalence), and non-beneficial examinations in patients classified as “Do not resuscitate” (33%–50% prevalence).^13^

The literature is scarce on research emphasizing patients who choose invasive treatments despite the low chance of benefitting them,^14^ as we noticed in our trial results. Kobewka et al.^14^ analyzed the end-of-life decisions of 13 patients with advanced organ failure diseases or at high risk of death who requested CPR if their heart stopped. In this trial, all 13 patients had previously seen a decision-aid video regarding CPR, its benefits, and harms, and still chose to ask for resuscitation maneuvers. The main reasons for their answers reflected a solid will to prolong life and the sense that refusing CPR meant choosing to die. Similar to this study, most participants lacked sufficient information on CPR and its consequences, and still, they defended this choice as essential and “worth a try.”^14^ The authors also highlight the high risk of discordance between the patient’s preferences and the performed treatments, classifying it as a seriously wrong event that must be prevented.

According to Portuguese law,^3^ the preferences stated on the Living Will must be respected by health professionals, with a few exceptions (patients no longer want the registered choices; decisions are outdated considering the scientific evolution; the circumstances that the patient predicted have changed). However, Portuguese law also states that the Advance Directives are invalid when against the law and public order, against good medical practices, or when their accomplishment might induce a non-natural and avoidable death.^3^

In this context, particularly in patients with progressive and incurable diseases receiving palliative care, clinicians have a moral obligation not to initiate ineffective treatments and ensure that both patients and families understand this concept of medical futility and maleficence to prevent future circumstances of displeasure and litigation.

Studies^15,16^ have explained that patients and their families must be involved in the decision-making process to respect their autonomy. However, this does not give them the right to receive or demand any desired treatment, as patients may have unrealistic goals for their end-of-life healthcare.^15,16^

The Advance Directives documents are considered a prospective consent form regarding the treatments for the end-of-life period.^17^ Some authors^10^ describe this document as a “proactive, informed refusal of therapies in a future state of incapacity.”^10^

Therefore, as stated by Beauchamp,^18^ crucial elements must be considered when an informed consent form is requested and signed. First, citizens must be competent to decide and have the capacity to receive and understand all information regarding the subject. Then, after receiving and integrating the available information, the citizen must be able to decide voluntarily and, finally, consent to the proposed treatment.^18^ In Portugal, citizens and patients can register their Advance Directives without mandatory medical counseling or health care assistance, although some citizens might request it. Consequently, one of the most critical elements of informed consent might not be fulfilled, as we cannot guarantee that citizens have complete knowledge and comprehension of the available healthcare treatments, indications, contraindications, and potentially harmful side effects before registering their Living Will. This assumes particular interest in palliative care patients, as the risk of therapeutic futility is considerable.

The Portuguese Medical Association document on patient rights and duties^19^ clearly states that patients can decide, in a free and informed way, whether to accept or refuse any treatment according to their self-determination rights. However, this document does not provide patients the right to choose treatments without benefits in their clinical situation.^19^ The Advance Directives’ document^4^ should be equally clear and not conducive to misinterpretations. These documents should include an explanatory section on the therapeutic futility and elucidate the patients on this subject.

Most legalized Advance Directives in European countries^20^ refuse supportive treatment and treatment limitations. In Portugal and many other countries in Europe, the documents are legally binding, and their content must be respected by healthcare teams, whereas in other countries, the documents are merely informative and indicative of the patient’s preferences.^20^

Many Advance Directives formularies, in countries such as Canada, England, USA, Spain, Germany or Australia, have an explanatory introduction regarding their content and its purpose.^21-26^ However, they lack information regarding the concept of futility and non-beneficial treatments and give the citizens the option to choose or not invasive treatments that might be considered futile.^21-26^ Nevertheless, some countries legalized Advance Directives documents that are more objective and less prone to misunderstandings by focusing citizens’ choices on refusing invasive and potentially harmful treatments instead of demanding treatments that might be considered futile.

In the Netherlands, the legalized Advance Directives^27^ only include a “do not resuscitate order” or a “written treatment prohibition and a request for euthanasia,” and the Finnish Advance Directives’^28^ formulary consists of a pre-written text refusing invasive treatments and demanding the interruption of previously started treatments if they are later recognized as futile.^28^

The Swiss Advance Directives’^29^ document, despite allowing citizens to choose invasive treatments for their end-of-life care, stresses that when citizens choose invasive treatments, they must accept the restrictions associated with the desire to stay alive. Citizens and patients can decide between “do not treat” or “treat as clinically indicated, even in cases of poor prognosis” and implicitly acknowledge that if any treatment is to be done, it must be clinically indicated.^29^

In France, the Advance Directives’^30^ document sends a more subtle message, which may reduce the likelihood of patients demanding non-benefitting treatments, because the document only allows patients to accept or refuse treatments. The use of these particular words (“accept and refuse”) has broader and deeper consequences than it seems at first sight, insofar as it implies that the treatments must be offered or proposed so that citizens can accept or refuse them. Therefore, if the medical team considers that a treatment is not beneficial and does not propose it to the patient, the patient has no legal way to request it and go against the good medical practice, as they can only “accept or refuse” treatments.^30^

Congruent with other countries, the primary purpose of Portuguese law^3^ is to allow citizens to refuse invasive and futile treatments such as non-benefiting reanimation or vital organ support. However, the document might allow a subversive interpretation and convey the idea that citizens can ask for any treatment, even those who might not benefit from them^3,4^

Even among medical teams, the futility concept is hard to define and recognize;^12^ therefore, we cannot expect patients to consider futility issues when choosing treatments for their end-of-life care if they are unfamiliar with the subject.

Physicians must be clear and honest when informing patients and caregivers about their clinical situation and prognosis, the treatments that can benefit them, their side effects, and possible influence on their quality of life so that their decisions are made with full conscience.^31^

Citizens and patients must be informed of their right and autonomy to accept or refuse the treatments proposed for their end-of-life care. They must be encouraged to analyze and question their treatment choices and discuss these issues with their loved ones. However, the futility issues must not be left out of the conversations, and the benefits of the good medical practices must be overvalued. These different concepts must be addressed and exhaustively discussed among patients, caregivers, and health professionals to improve their knowledge of the subject. Particularly in palliative care, as patients face progressive and terminal diseases and have poor benefits from curative treatments, discussion of the futility concept must be considered a priority by healthcare teams.^32^

Therefore, good communication habits between patients, families, and healthcare teams are vital for clarifying patients’ preferences for their end-of-life period. In addition, reducing their pretension of being subjected to treatments that might be harmful must be a fundamental goal to achieve.^10^

## CONCLUSION

The Advance Directive legislation aimed to promote the patient’s autonomy and self-determination in refusing invasive and futile treatments that might not benefit them as scientific knowledge is evolving.^5^

However, as observed in one group of 60 patients and 60 caregivers, many participants chose invasive treatments and artificial organ support for end-of-life care (23%-40%). Although these treatments may be considered futile in the palliative patient group, none of the participants were familiar with this concept or definition.

Healthcare teams have a moral duty to elucidate to patients and caregivers about the futility theme and must consider it a priority when patients face progressive and terminal diseases, defend the patients’ best interests, and ensure that their decision-making is conscientious and well-founded.^31^

We advocate that every citizen and patient who manifests the will to register their Advance Directive must be informed by their healthcare physician about their clinical scenario and prognosis, the concept of therapeutic futility, and harmful treatments before filling the document. They should also be informed about the Advance Directives^4^ content, its limits, and the circumstances that might question its validity.

Caregivers should also be involved in the decision-making process to help clarify the patients’ wishes as legitimate surrogates.^33^

However, the Portuguese Advance Directive document^4^ as a legal instrument that empowers patients to exercise their autonomy and that the healthcare team must respect, should be transparent and not prone to misinterpretations, not to give rise to legal issues and disputes. Although in Portuguese law on Advance Directives, the right to refuse treatment is absolute and the right to request is not compulsory, good medical practice must be respected and achieved.

We consider that the Advance Directive document^4^ should have an explanatory section that elucidates citizens and patients’ rights to accept or refuse the treatments that they are being offered, but should also clearly mention that they must not demand treatments that have no benefit and are considered futile in their clinical situation, as most probably will not receive them.

We propose a simple change of the words that precede section 2 of the Portuguese Advance Directives’ document “Healthcare to Receive / Not to Receive,” to “Healthcare to Accept / Refuse,” as this statement preceding the Advance Directives’ questions, will reinforce the idea that treatments must be offered so that the citizens have the right to accept or refuse them.

This subject must also be continuously debated among healthcare professionals, in conferences, meetings, and day-to-day ordinary clinical practice to facilitate its recognition, definition, and worldwide discussion.

We strongly believe that improving citizens’, patients, and caregivers’ health literacy might reduce their probability of choosing futile treatments and avoid misinterpretations and false expectations when completing Advance Directive documents. Healthcare personnel who are familiar with these concepts and the patient’s medical history, family members, and social environment, should have a prominent place in this accomplishment, mediating the decision-making process and promoting a therapeutic strategy consistent with the patients’ wishes and good medical practices.
